# A primer on the mouse basal body

**DOI:** 10.1186/s13630-016-0038-0

**Published:** 2016-04-25

**Authors:** Galo Garcia, Jeremy F. Reiter

**Affiliations:** Department of Biochemistry and Biophysics, Cardiovascular Research Institute, University of California, San Francisco, San Francisco, CA 94158 USA

**Keywords:** Basal body, *Mus musculus*, Transition fiber, Distal appendage, Subdistal appendage, Basal foot, Rootlet, Daughter centriole, Primary cilium, Motile cilium

## Abstract

The basal body is a highly organized structure essential for the formation of cilia. Basal bodies dock to a cellular membrane through their distal appendages (also known as transition fibers) and provide the foundation on which the microtubules of the ciliary axoneme are built. Consequently, basal body position and orientation dictates the position and orientation of its cilium. The heart of the basal body is the mother centriole, the older of the two centrioles inherited during mitosis and which is comprised of  nine triplet microtubules arranged in a cylinder. Like all ciliated organisms, mice possess basal bodies, and studies of mouse basal body structure have made diverse important contributions to the understanding of how basal body structure impacts the function of cilia. The appendages and associated structures of mouse basal bodies can differ in their architecture from those of other organisms, and even between murine cell types. For example, basal bodies of immotile primary cilia are connected to daughter centrioles, whereas those of motile multiciliated cells are not. The last few years have seen the identification of many components of the basal body, and the mouse will continue to be an extremely valuable system for genetically defining their functions.

## The organism: *Mus musculus*

The house mouse *Mus musculus* is the vertebrate most widely used in biomedical research. The long history of mouse genetics, building off of the work of Victorian mouse fanciers and amplified by the development of both forward and reverse genetic approaches, has provided a rich, tractable and powerful set of genetic tools in mouse [1]. As we share 99% of our genes with mice, they are useful for modeling many aspects of human basal body function. For example, some ciliopathies, such as primary ciliary dyskinesia (PCD) and Meckel syndrome (MKS), are well modeled by mouse mutations in orthologous genes [2–4]. However, other ciliopathies such as nephronophthisis (NPHP) and Joubert syndrome (JBTS) are imperfectly recapitulated with existing mouse mutations [5–9]. As the ability to humanize portions of the mouse genome and create tailored mutations increases, it may be possible to more accurately model complex phenotypes related to cilia and basal bodies.

## Murine basal body structure

### Murine basal bodies contain triplet microtubules

The nine triplet microtubules that make up the barrel of the basal body are named A, B, and C from internal to external. As with all centrioles, the plane of the triplet microtubules is tilted such that the vector from the A-tubule to the C-tubule, if viewed from the proximal end of the centriole, points counterclockwise [10, 11]. The doublet microtubules of the ciliary axoneme are contiguous with the basal body A- and B-tubules, whereas the C-tubule terminates within the distal centriole or in a region between the basal body and the cilium called the transition zone [12–16].

### Different murine cell types display different basal body architectures

Distinct types of mouse cells possess structurally and functionally distinct types of cilia. The principal types of cilia are immotile primary cilia that can interpret intercellular signals, highly modified signaling cilia such as the photoreceptor connecting cilium, motile nodal cilia involved in left–right axis determination, immotile multicilia of the olfactory receptor neurons, motile multicilia that move fluid in the lung, brain ventricles and fallopian tubes, and the sperm flagellum.

While it is unclear whether basal bodies of distinct types of cilia contain sets of proteins unique to that ciliary type, the structure of the cilium itself can vary in ways that may be dependent on the basal body [17]. One example is the transition zone, a region between the basal body and cilium characterized by Y-fibers connecting the microtubules to the ciliary membrane. The transition zone can be short, such as in fibroblasts, or long, such as in photoreceptors.

Many motile cilia, such as those of tracheal and ependymal cells, have a central pair of microtubules in addition to the nine doublets of the axoneme (the so-called 9 + 2 arrangement of microtubules). Mutations in mouse *Hydin*, which encodes a protein associated with the central pair microtubules, causes defects in ciliary bending and beat frequency, suggesting that the central pair is critical for normal ciliary motility [18, 19].

However, not all motile cilia have a central pair. For example, most nodal cilia lack the central pair [20]. Consistent with the absence of the central pair in nodal cilia, human *HYDIN* mutations affect the motility of the cilia of the respiratory tract but do not cause left–right axis defects [21]. Conversely, not all 9 + 2 cilia are motile. Olfactory sensory neurons possess 9 + 2 cilia, but not the dynein arms required for ciliary motility [22].

Unlike the peripheral axonemal microtubules, the central pair microtubules are not continuous with microtubules of the basal body: they arise in the transition zone distal to the basal body. How the basal body influences whether the axoneme possesses the central pair remains unclear, but, at least in invertebrates, central pair formation depends on basal body components such as BLD10/CEP135 [17].

### The mouse δ- and ε-tubulin genes

Consistent with the proposed link between δ- and ε-tubulin and the presence of triplet microtubules in centrioles, the mouse genome contains orthologs of the genes encoding δ- and ε-tubulin, *Tubd1* and *Tube1* [23, 24]. In *Chlamydomonas,* δ-tubulin is essential for the production of two and only two flagella and the production of triplet microtubules in the basal body: mutants lack the C-tubule [25]. *Chlamydomonas * ε-tubulin is critical for basal body formation or maintenance and is required for the formation of both basal body doublet and triplet microtubules [26]. In mammalian cells, δ-tubulin localizes to the spindle poles and co-immunoprecipitates with γ-tubulin, and ε-tubulin localizes to the subdistal appendage of the basal body [27, 28]. Answering the question of whether the function of mouse δ- and ε-tubulin is similar or distinct from that in *Chlamydomonas* awaits functional genetic analysis.

### Accessory structures of mouse basal bodies

Murine basal bodies are accompanied by, depending on the phase of the cell cycle and cell type, either no (in the instances of sperm and multiciliated cells), one (monociliated cells in G1, G0, or early S phase), or three (monociliated cells in late S or G2 phase) centrioles [29, 30]. During G1 phase of most ciliated cells, the proximal end of the basal body is connected to the proximal end of the daughter centriole by a linkage, and the daughter centriole is oriented roughly orthogonally to the basal body [31]. In contrast, the basal bodies of motile multiciliated cells are not physically associated with daughter centrioles, although the daughter centrioles do have crucial roles in the generation of the many basal bodies possessed by these cells [32].

Basal bodies are surrounded by pericentriolar material. The pericentriolar material is comprised of proteins such as Pericentrin, appears moderately electron dense by EM, and nucleates the minus ends of many cytoplasmic microtubules [33–35]. On the periphery of the pericentriolar material exist large electron-dense protein complexes called centriolar satellites involved in ciliogenesis and centriole duplication [36–38].

### Mouse basal body appendages

Murine basal bodies possess a variety of appendages, including a rootlet, distal appendages, and subdistal appendages or a basal foot. Indeed, the basal body is distinguished from daughter centrioles and procentrioles by the presence of these appendages. The relationship of subdistal appendages to the basal foot is unclear. Both project from the sides of the basal body at nearly the same position, approximately 350 nm from the proximal end of the basal body, and both are associated with microtubule nucleation [39, 40]. Basal bodies have up to nine subdistal appendages, but only one or two basal feet. The basal foot further differs from subdistal appendages in that it is larger and is more electron dense. Subdistal appendages and basal feet are mutually exclusive and have some of the same genetic requirements, suggesting that subdistal appendages may coalesce to form the basal foot [41]. During G2 phase, the subdistal appendages or basal foot are lost and do not reappear until the next G1 phase [42, 43].

The nine distal appendages project outward from the distal end of the basal body and are required for membrane docking and ciliogenesis [44–46]. Once the basal body docks to a membrane, distal appendages are often referred to as transition fibers. The distal appendages possess Cep164, Cep89, Cep83, Fbf1, and Sclt1, and all five are involved in ciliogenesis, with Cep83 being specifically important for membrane docking [45, 46].

The rootlet is a thick (80–100 nm) striated bundle of filaments that projects from the proximal end of the basal body and extends close to the nucleus [47]. Striations orthogonal to the filament axis are present at intervals of ~55–75 nm [47]. Rootlets are associated with basal bodies of both motile and immotile cilia, such as photoreceptor cells. In this cell type, the rootlet extends from the outer segment, a highly specialized modified cilium, to the synaptic terminal at the opposite end of the cell [48, 49]. One component of the rootlet is Rootletin [47, 50]. Consistent with the rootlet being dispensable for ciliary motility and signaling, a mutation in mouse *Rootletin* (also known as *Crocc*) that disrupts rootlet formation does not abrogate ciliary beating or phototransduction [51]. However, this mutation causes photoreceptor degeneration and may reduce mucociliary clearance, suggesting that mechanical support provided by the rootlet is essential for the long-term maintenance of ciliary function [51, 52].  

In addition to its function in the rootlet, Rootletin, together with C-Nap1, forms fibers that connect mother and daughter centrioles and may function in centrosome cohesion [31, 53, 54]. Mutations in the human homolog of *C*-*Nap1*, called *CEP250 or CEP2*, are associated with Usher syndrome, a disease characterized by retinitis pigmentosa and hearing loss [55]. In cattle, mutations in *C*-*Nap1* are associated with microcephaly, suggesting that the linker between mother and daughter centrioles plays important roles in neural development in mammals [56].

### Noteworthy EM studies of mouse basal bodies

**Fig. 1:**
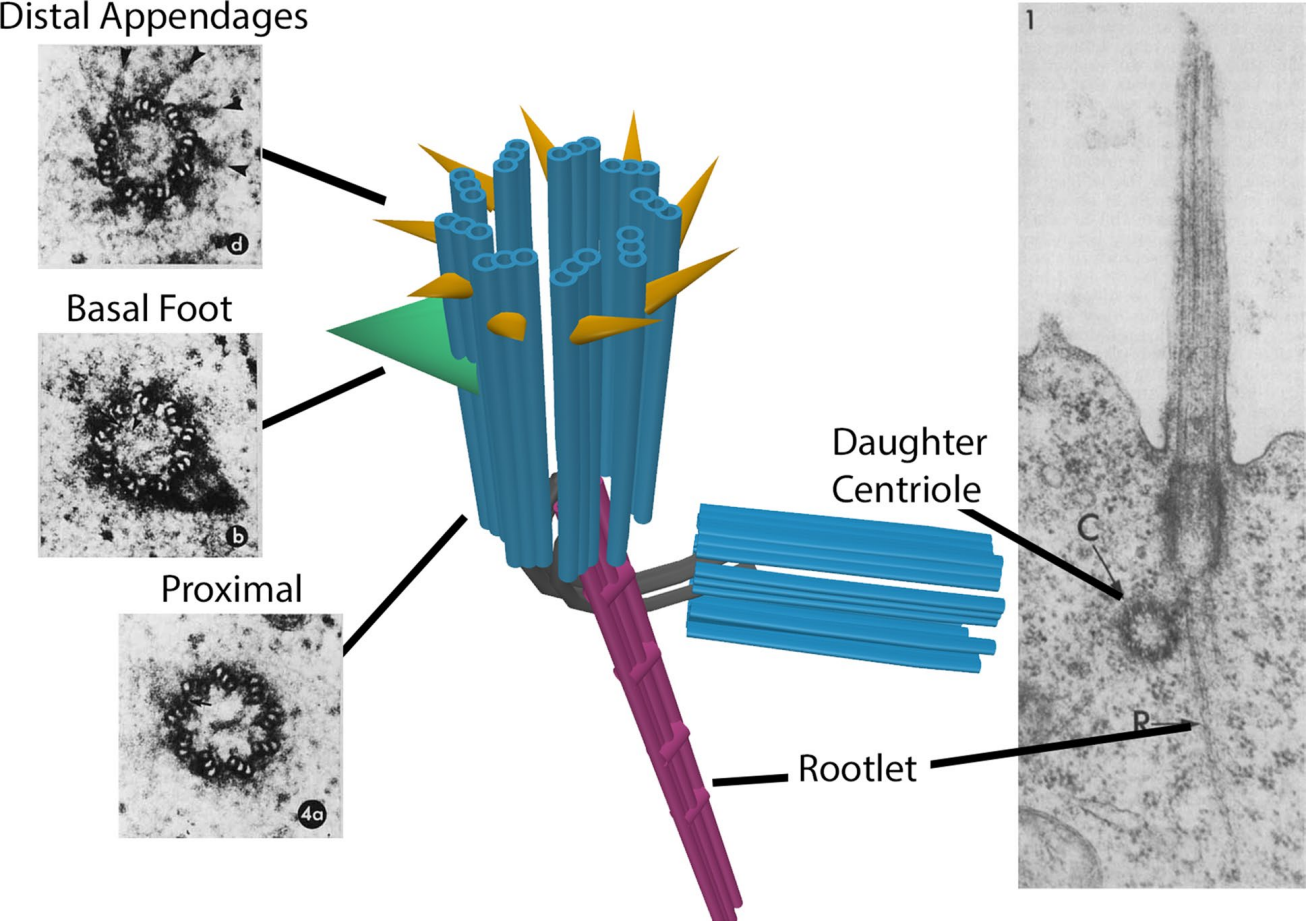
Basal body ultrastructure. At the distal end of the basal body, distal appendages or transition fibers are blades that have ninefold symmetry and radiate outward from the triplet microtubules. Proximal to the distal appendages is the basal foot, an electron-dense and cone-shaped structure projecting from one side of the basal body. Some basal bodies have multiple basal feet. The proximal end of the basal body is attached to the striated rootlet and to filaments that connect the basal body to the proximal end of the daughter centriole. Motile cilia in multiciliated cells lack associated daughter centrioles. Image credits: serial-section electron micrographs of transition fibers, the basal foot, and proximal basal body were originally published in [44]. Electron micrograph illustrating the striated rootlet and the daughter centriole was originally published in [14]

Many investigators have analyzed both rodent and other vertebrate basal bodies, thereby revealing that the basal bodies of vertebrates are highly similar. For example, Sergei Sorokin described the formation of primary cilia in rat tissue and organ cultures of chicken duodenum [57]. In addition, he and Ronald Gordon defined the ultrastructure of motile cilia in the rat lung [58, 59]. Ellen Dirksen examined the structure of basal bodies in the mouse fallopian tube [60]. Wilsman et al. [44] performed serial EM studies of primary cilia in chondrocytes. The micrographs of serial sections in chondrocytes show with remarkable clarity the relative orientation of the transition fibers, the basal foot, and the triplet microtubules. More recently, Kazuhiro et al. performed electron tomographic studies demonstrating the role of Odf2 in the formation of distal and subdistal appendages [39]. The electron tomograms nicely show the ultrastructure of the basal body appendages in three dimensions (Fig. 1).

### Early studies identifying basal body features

The basal foot was described in 1954 by Fawcett and Porter as “a small process” extending from the anterior face of the amphibian basal body [61]. In rodents, Rhodin and Dalhamn in 1956 identified the basal foot as a “cytoplasmic process” on basal bodies in rat tracheal epithelial cells [62]. By 1961, the term basal foot was used by Gibbons in his studies of cilia in the gills of the freshwater mussels [10]. In mouse embryonic fibroblasts, Wheatley identified the basal foot in 1969 [63].

The transition fibers were identified later than the basal foot. In 1972, Anderson identified transition fibers in mammalian fallopian tube cells, referring to them as “alar sheets” [64]. Surprisingly, the ciliary rootlet was identified before the advent of electron microscopy: Friedreich and Engelmann identified the ciliary rootlet using histological methods in the nineteenth century [61, 65, 66].

## Origins of mouse basal bodies

Most basal bodies of cycling mouse cells, including many cells with primary cilia, are derived from mother centrioles inherited during mitosis [67]. Most basal bodies of non-cycling multiciliated cells are built from an electron-dense organelle called the deuterosome [32, 68]. Interestingly, it is the daughter centriole, not the mother centriole, that contributes to formation of the deuterosome [32].

In stark contrast to later phases of development, mouse cells lack centrioles during the first few cleavages following fertilization [69]. Despite the absence of centrioles, these early mouse blastomeres form and organize microtubules [70]. The pericentriolar material unassociated with centrioles may serve as the source of the MTOC activity in these cells [71, 72]. In particular, Plk4 and Cep152 can localize to an acentriolar MTOC to help organize microtubules [73].

Centrioles do not arise during development until the early blastocyst stage, indicating that these embryonic mouse cells must build centrioles de novo [74, 75]. The de novo synthesis of centrioles in human cells is error prone, suggesting that the cell’s ability to construct a structurally accurate centriole may be facilitated by the existence of a pre-existing centriole [76]. Loss of mouse Sas4 (also called Cenpj) disrupts formation of centrioles, basal bodies, and cilia, but does not halt cell cycle progression or embryonic development until midgestation, indicating that, despite their genesis early in development, they are not essential for some forms of cell division [77].

### During development and in adult tissues, all ciliated cells have basal bodies

In cycling cells, the basal body becomes a mother centriole after the cilium is disassembled. This former basal body serves as a part of a spindle pole during mitosis, and thus mitosis can be considered a phase of the cell cycle during which basal bodies do not exist. Although mouse cells disassemble their cilia before entering mitosis, a heterozygous mutation in *Pifo* can cause mouse cells to retain a ciliary remnant into mitosis (although it may no longer be physically associated with the basal body) and, perhaps consequently, can cause mitotic defects [78]. Multiciliated cells are terminally differentiated and thus possess basal bodies during their entire lifetimes [79].

## Basal body contribution to microtubule-organizing center (MTOC) activity

In addition to supporting ciliogenesis, the basal body contributes to MTOC activity although, as mentioned above, it is not essential for MTOC activity. Many microtubules are anchored in the pericentriolar material itself, but the subdistal appendages and subdistal appendage proteins, such as Ninein and the Dynactin complex, are also implicated in anchoring microtubules [35, 80–85]. How the function of microtubules originating from the pericentriolar material and those attached to the subdistal appendages or basal foot differs will be interesting to determine.

## Identification of mouse basal body components

A handful of proteomics and genomics screens have identified many mouse basal body components and identified many other candidate components. A transcriptomic study of mouse tracheal epithelial cells (mTECs) during ciliogenesis has identified more than 600 genes that are upregulated during early ciliogenesis [86]. Reflecting the genesis of both basal bodies and cilia during mTEC differentiation, these upregulated genes include those that encode ciliary and basal body proteins, and thus has been a boon for the identification of basal body components.

To identify genes involved in basal body and ciliary biology, targeted and genome-wide knockdown screens have been performed in mouse cells that possess primary cilia [87, 88]. Knockdown of these genes, or orthologous genes in human cells, can result in a variety of phenotypes, including loss of cilia, short cilia, long cilia, as well as ciliary transport defects in the absence of obvious structural defects [87–89]. The cell biological origin of these phenotypes and whether the associated gene products act at the cilium, the basal body, or elsewhere remains to be elucidated in most cases.

The proteome of the mouse photoreceptor sensory cilium complex, an isolated preparation containing the axonome, the basal body, and the ciliary rootlet of the photoreceptor outer segment, has identified over 1000 candidate ciliary proteins [90]. In addition to studies in mouse cells, proteomic and genomic screens have identified novel basal body and ciliary components in human and rat cells [91–93]. For example, mass spectrometry-based proteomics analysis of centrosomes and centrosomal protein interactors has identified novel centrosomal proteins, many of which have functions relevant to basal bodies [94–97]. Given the close evolutionary relationship between mice and other mammals, the mouse orthologs of the proteins identified in such screens are likely to inform mouse basal body biology.

Two independent comparative genomics studies focused on identifying genes involved in ciliary biology. Comparison of the gene complement of unciliated organisms with those of ciliated organisms identified genes involved in ciliogenesis and ciliary function [91, 98]. Although these computational approaches do not discriminate between genes encoding basal body and ciliary proteins, a subset is likely to encode components of the basal body. For example, both studies implicated the basal body component Sas4 as being specific to ciliated organisms.

## Notable basal body findings made using mice

Genetic studies in mice have been especially useful in determining the physiological functions of basal bodies. In most instances, a single mutant allele exists, providing important but limited insight into basal body function. In select cases, an allelic series provides more nuanced insight into the full range of basal body functions. For example, a hypomorphic mutation indicates that Odf2 mediates the orientation of basal feet, and proper polarization of basal feet is required for polarity of the ciliary beat in tracheal epithelial cells [99]. A stronger allele reveals that Odf2 is also essential for formation of the transition fibers and basal feet [39].

Like Odf2, Chibby homolog 1 (Cby1), a protein that localizes to the distal centriole, aids in docking of the basal body to the plasma membrane and is essential for mucociliary clearance in the airway epithelium [100–102]. One important function of Cby1 is in the recruitment of Ahi1 to the transition zone [101]. Understanding how Cby1, Odf2, and other basal body proteins orient the basal body to provide effective mucociliary clearance will provide insights into how ciliary orientation and motive force are achieved.

Other studies have helped illuminate how the distal centriole functions in ciliogenesis. For example, genetic and cell biological studies on mouse C2cd3 have demonstrated that it localizes to centriolar satellites, as well as to the distal end of centrioles, and that C2cd3 is required for formation of the distal appendages and for ciliary vesicle docking to the mother centriole [103, 104]. Loss of C2cd3 blocks removal of Cp110 from, and recruitment of Ttbk2, to the mother centriole, early steps in the initiation of ciliogenesis [104, 105]. Loss of C2cd3 also blocks recruitment of Ift88 and Cep164 to the distal appendage of the mother centriole [104]. Mouse *C2cd3* mutants display phenotypes reminiscent of human ciliopathies, including severe polydactyly, situs defects, and disruption of the dorsal–ventral patterning of the neural tube [106]. Hedgehog signaling is disrupted in these mice, reflecting the essential function for cilia in transducing vertebrate Hedgehog signals [106]. Ofd1, a protein mutated in oral-facial-digital syndrome, co-localizes with C2cd3 at the distal centriole [103]. Whereas depletion of C2cd3 leads to shorter centrioles and the loss of the distal appendages, mutation of Ofd1 leads to centriole hyperelongation [103].

Another protein that localizes to the distal end of centrioles, Talpid3, interacts with Cp110 and regulates ciliogenesis [107–109]. *Talpid3* mutant mice lack primary cilia, and have situs, neural tube, and facial defects [107, 110]. Taken together, these results indicate that the distal centriole appears to be an important locale where a complex of proteins coordinates with Cp110 to initiate ciliogenesis.

EHD1 and EHD3 are yet additional distal centriole proteins that are required for ciliary genesis. EHD1 is involved in ciliary vesicle formation and the removal of Cp110 [111, 112]. Future investigation of potential centriole “capping” proteins, as well as other proteins that regulate centriole length, will help reveal how architecture varies in different cell types to promote the diverse functions of the basal body.

Genetic studies have the advantage of having the capacity to identify regulatory inputs that do not directly involve basal body components, or even protein-coding genes. For example, the microRNAs miR-34/449 may promote the biogenesis of motile cilia by repressing *Cp110* [113]. Consequently, mutant mice lacking these microRNAs are infertile and display defective mucociliary clearance [113].

## Strengths and future of basal body research in *Mus musculus*

Due to the organism’s genetic tractability, the mouse is the most commonly used experimental organism to study vertebrate development and to model human disease. One of the strengths of the mouse as an experimental organism is the ability to tailor the genome, a strength that is growing as a new generation of genetic tools becomes widely used. Genes required for basal body formation or function can be mutated, and phenotypes can be analyzed in a wide range of cell types with a wide variety of ciliary types, illuminating the function of basal bodies in development, physiology, and disease.

Another strength of the mouse as a model organism is the breadth of research tools available. For example, there are many antibodies available for the detection of basal body and ciliary proteins. A weakness of the mouse is the difficulty in acquiring sufficient material for some approaches, such as the proteomics of basal bodies in specific cell types.

Future prospects for research on basal bodies in the mouse are diverse. How is duplication of the basal body controlled in primary ciliated and multiciliated cells? What role do basal bodies have in transducing developmental cues, such as Hedgehog signals? How do basal bodies interact with the planar cell polarity pathway to control the orientation of the motile cilia that move external fluids? Genetic modeling of basal body-associated diseases, such as ciliopathies, in mice will continue to help identify the cell biological origins of human disease, but also will illuminate the diverse functions of basal bodies in fundamental cellular processes such as ciliogenesis, ciliary motility, centriole duplication, and microtubule organization.

## Abbreviations

MTOC: Microtubule organizing center
EM: electron microscopy
